# Impairment of Hepatic and Renal Functions by 2,5-Hexanedione Is Accompanied by Oxidative Stress in Rats

**DOI:** 10.1155/2014/239240

**Published:** 2014-10-15

**Authors:** Isaac A. Adedara, Amos O. Abolaji, Blessing E. Odion, Isioma J. Okwudi, Abiola A. Omoloja, Ebenezer O. Farombi

**Affiliations:** Drug Metabolism & Toxicology Research Laboratories, Department of Biochemistry, College of Medicine, University of Ibadan, Ibadan 20005, Nigeria

## Abstract

2,5-Hexanedione (2,5-HD) is the toxic metabolite of n-hexane which is widely used as solvent in numerous industries. The present study elucidated the precise mechanism of 2,5-HD in hepatorenal toxicity by determining the involvement of oxidative stress in rats. Adult male Wistar rats were exposed to 0, 0.25, 0.5, and 1% 2,5-HD in drinking water for 21 days. Exposure to 2,5-HD caused liver and kidney atrophy evidenced by significant elevation in serum aminotransferases, alkaline phosphatase, albumin, bilirubin, urea, creatinine, and electrolytes levels compared with control. The marked dose-dependent increase in total cholesterol (TC), triglyceride (TG), and low-density lipoprotein (LDL) was accompanied with significant decrease in high-density lipoprotein (HDL) levels in 2,5-HD-exposed animals when compared with the control. Administration of 2,5-HD significantly diminished glutathione (GSH) level but increased the activities of superoxide dismutase (SOD), catalase, glutathione peroxidase (GPx), and glutathione-S-transferase (GST) concomitantly with marked elevation in hydrogen peroxide (H_2_O_2_) and malondialdehyde (MDA) levels in liver and kidney of the treated groups compared with control. These findings suggest that undue exposure to 2,5-HD at environmentally relevant levels may impair liver and kidney functions through induction of oxidative stress.

## 1. Introduction

2,5-Hexanedione (2,5-HD) is the main toxic metabolite of n-hexane, an organic solvent widely used in chemical engineering and pharmaceutical and cosmetic industries [1]. Previous toxicological studies have shown that n-hexane and its metabolites, 2,5-HD and 2-hexanone, were detected in the liver, kidney, brain, blood, and the developing fetus at time points up to 18 hours after exposure [2]. 2,5-HD is known to cause testicular dysfunction, neurotoxicity, and genotoxicity and it adversely affects liver and kidney function in animals and humans [3–6]. An uncontrolled exposure to toxic industrial solvents is capable of causing biological damage which eventually could lead to pathological conditions and organ damage [7]. There is a growing concern over the safety of 2,5-HD exposure in humans. Epidemiological studies have been reported on n-hexane toxicity in Taiwan and China [1, 8]. However, relatively little is known about the mechanism of 2,5-HD toxicity to the liver and kidney.

The adverse effects resulting from exposure to xenobiotics could occur via several mechanisms leading to significant alterations in the levels of biomolecules such as enzymes and metabolic products, normal functioning, and histomorphology of the organs. The paucity of information in the literature about 2,5-HD-induced hepatorenal toxicity accentuates the need to undertake a detailed study evaluating the antioxidant status of the kidney and liver in rats exposed to 2,5-HD. It is well known that oxidative stress is involved in the pathogenesis of several diseases following exposure to environmental contaminants. The liver is particularly vulnerable to toxicity produced by reactive metabolite because it is the major site of xenobiotic metabolism. The kidney is a highly specialized organ that maintains the internal environment of the body by selectively excreting or retaining various substances according to specific body needs [9]. The ability of the kidney to concentrate the tubular fluid by removing water and salts, consequently, predisposes the kidney to toxic chemicals. The biotransformation of chemicals to reactive and potentially toxic metabolites is a key feature of hepatic and renal toxicity [10]. The antioxidants status in humans reflects the dynamic balance between the antioxidant defense and prooxidant conditions and has been suggested as a useful tool in estimating the risk of oxidative damage [11, 12].

To delineate the mechanism of action of 2,5-HD at subcellular levels in liver and kidney, we investigated its effect on the antioxidant enzymes, oxidative stress indices, and biomarkers of renal and hepatic functions following subchronic exposure in the treated rats.

## 2. Materials and Methods

### 2.1. Chemicals

2,5-Hexanedione (98.99%), epinephrine, glutathione (GSH), 5,5′-dithio-bis-2-nitrobenzoic acid (DTNB), hydrogen peroxide (H_2_O_2_), thiobarbituric acid (TBA), and 1-chloro-2,4-dinitrobenzene (CDNB) were purchased from Sigma Chemical Co. (St. Louis, MO, USA). All other reagents were of analytical grade and were obtained from the British Drug Houses (Poole, Dorset, UK). Kits for serum biochemistry were purchased from Randox Laboratory Limited, United Kingdom.

### 2.2. Animal Model and Experimental Protocol

A total of thirty-two healthy adult male Wistar rats (10 weeks old, ≈170 g) purchased from the Department of Biochemistry, University of Ibadan, Ibadan, Nigeria, were used for this study. They were housed in plastic cages placed in a well-ventilated rat house, provided rat pellets and water* ad libitum*, and subjected to natural photoperiod of 12 hr light: 12 hr dark. All the animals received humane care according to the criteria outlined in the “Guide for the Care and Use of Laboratory Animals” prepared by the National Academy of Science and published by the National Institute of Health [13]. The experiment was performed according to the guidelines and approval of institutional animal ethics committee. The rats were randomly divided into four groups of eight rats per group. The animals in the control group received distilled water alone for 21 days whereas the remaining groups were exposed to 0.25%, 0.5%, and 1% 2,5-HD, respectively, in drinking water according to established protocol [14] for 21 days.

All the rats were sacrificed by cervical dislocation 24 hours after the last intake of 2,5-hexanedione, and blood was collected by cardiac puncture. The livers and kidneys were quickly removed, weighed, and placed on an ice bath. The blood was allowed to clot and centrifuged at low speed (3000 ×g) at room temperature for 15 minutes. The body weights of rats were taken before exposure to various treatments and before killing.

### 2.3. Serum Biochemistry

Serum activities of aspartate aminotransferase (AST) and alanine aminotransferase (ALT) were determined by the method of Reitmann and Frankel [15]. Alkaline phosphatase (ALP) was determined according to the recommendation of German Society of Clinical Chemistry (Rec. GSCC) [16]. The levels of serum urea, creatinine, and bilirubin were estimated by Fawcett and Scott [17], Henry [18], and Jendrassik and Grof [19], respectively. Serum electrolytes (sodium, potassium, chloride, and bicarbonate ions) were determined by flame photometry.

### 2.4. Metabolic Parameters

Levels of serum total cholesterol (TC), triglyceride (TG), low-density lipoprotein (LDL), and high-density lipoprotein (HDL) were determined using commercially available diagnostic kits (Randox Laboratories Limited, UK).

### 2.5. Biochemical Assay

Remaining portions of liver and kidney were homogenized in 50 mM Tris-HCl buffer (pH 7.4) containing 1.15% potassium chloride. Following the centrifugation of the homogenate at 10,000 ×g for 15 minutes at 4°C, the supernatant was collected for the estimation of superoxide dismutase (SOD) activity by the method of Misra and Fridovich [20]. Catalase (CAT) activity was assayed by using H_2_O_2_ as the substrate according to the method of Clairborne [21]. Protein concentration was determined by the method of Lowry et al. [22]. Glutathione (GSH) level was estimated at 412 nm using the method described by Jollow et al. [23]. Activity of glutathione peroxidase (GPx) was determined by the method of Rotruck et al. [24]. Glutathione-S-transferase (GST) was assayed by the method of Habig et al. [25]. Hydrogen peroxide generation was assessed by the method of Wolff [26]. Lipid peroxidation was quantified as malondialdehyde (MDA) according to the method described by Farombi et al. [27] and expressed as micromoles of MDA per milligram protein.

### 2.6. Histopathology

Liver and kidney biopsies were processed for histology according to Songur et al. [28]. Briefly, liver and kidney specimens were fixed in 10% neutral-buffered formaldehyde solution. After dehydration procedures, the samples were blocked in paraffin. Sections of 4-5 *μ*m were cut by a microtome and stained with hematoxylin and eosin (H&E). All slides were coded before examination with light microscope and photographed using a digital camera by investigators who were blinded to control and 2,5-hexanedione-treated groups.

### 2.7. Statistical Analyses

Statistical analyses were carried out using one-way analysis of variance (ANOVA) to compare the experimental groups followed by Bonferroni's test to identify significantly different groups (SPSS for Windows, version 17). *P* < 0.05 was considered statistical significance.

## 3. Results

### 3.1. Body Weight and Organ Weights

The rats in all the experimental groups remained active and vigorous throughout the treatment period. The body weight and relative organ weights of control rats and those exposed to 2,5-HD are presented in Table 1. Following exposure period, the rats that were treated with 0.5% and 1% 2,5-HD showed a significant (*P* < 0.05) reduction in body weight when compared with the control. However, administration of 2,5-HD significantly increased the relative liver and kidney weights when compared with the control.

### 3.2. Repeated Exposure to 2,5-HD Impairs Liver Function

To investigate the impact of 2,5-HD treatment on liver function, the levels of AST, ALT, ALP, albumin, and total and conjugated bilirubin were determined in the serum of control and 2,5-HD-treated rats after 21 days.  Table 2 shows the effect of 2,5-HD on biomarkers of hepatic dysfunction in the treated rats. The results indicate a significant, dose-dependent elevation in AST, ALT, ALP, albumin, and total and conjugated bilirubin levels in rats exposed to 2,5-HD when compared with the control.

### 3.3. Exposure to 2,5-HD Alters Lipid Profile

Table 3 shows that administration of 2,5-HD resulted in considerable metabolic disorders in the treated rats. There was a dose-dependent significant decrease in HDL levels in all the animals administered with 2,5-HD when compared with control. However, there was a marked increase in the levels of LDL, TG, and total cholesterol in all 2,5-HD-treated animals when compared with the control.

### 3.4. Exposure to 2,5-HD Induces Renal Dysfunction

To investigate the integrity of the kidney following 2,5-HD exposure to rats, the concentrations of biomarkers of renal dysfunction were determined. The effects of 2,5-hexanedione on urea, creatinine, Na^+^, K^+^, HCO_3_^−^, and Cl^−^ levels in the treated rats are shown in Table 4. The result indicated that 2,5-HD treatment caused a significant, dose-dependent elevation in serum urea, creatinine, and all the electrolytes levels when compared with the control.

### 3.5. Hepatorenal Effect of 2,5-HD Is Mediated by Increased Oxidative Stress

**Figure 1:**
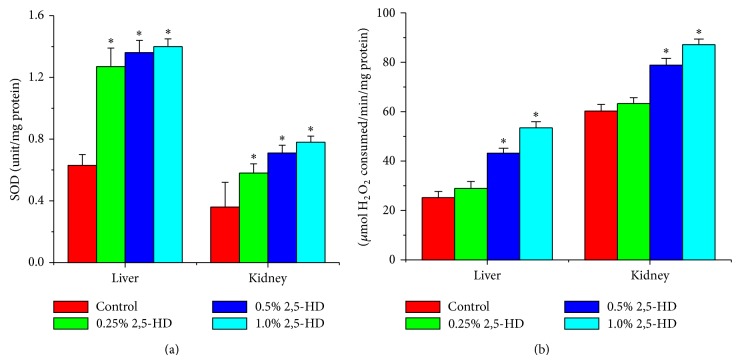
Comparison of hepatic and renal SOD and CAT activities among the control and treated rats (*N* = 8). Values represent mean ± SD of enzyme activity (unit/mg protein of tissue). Asterisk indicates statistical difference from control (*P* < 0.05).

**Figure 2:**
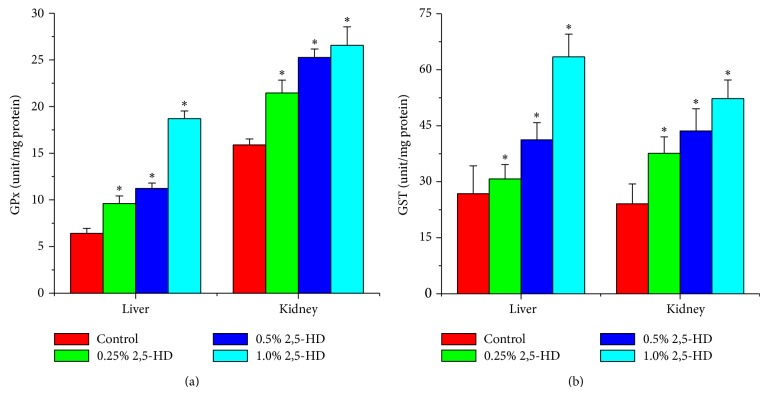
Comparison of hepatic and renal GPx and GST activities among the control and treated rats (*N* = 8). Values represent mean ± SD of enzyme activity (unit/mg protein of tissue). Asterisk indicates statistical difference from control (*P* < 0.05).

**Figure 3:**
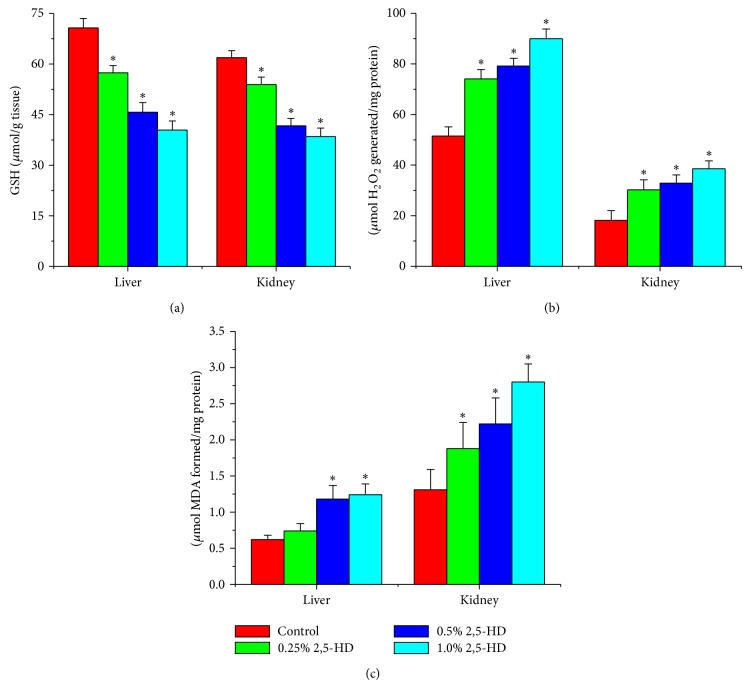
Comparison of hepatic and renal GSH, H_2_O_2_ generation, and MDA levels among the control and treated rats. Data are described as mean ± SD of eight animals per group. Asterisk indicates statistical difference from control (*P* < 0.05).

Following exposure to 2,5-HD, the antioxidant statuses of the liver and kidney of the treated rats were determined using a panel of assays including enzymatic and nonenzymatic antioxidant levels along with hydrogen peroxide and lipid peroxidation levels. Figures 1, 2, and 3 show the effects of 2,5-HD on the hepatic and renal antioxidant status in the experimental animals. Repeated exposure to 2,5-HD caused a significant (*P* < 0.05) increase in the activities of antioxidant enzymes SOD and CAT in both liver and kidney of all the treated groups when compared with the control animals. Moreover, the significant increases in the activities of GPx and GST were accompanied by a significant decline in the levels of GSH in both hepatic and renal tissues when compared with the control. However, H_2_O_2_ generation and MDA levels, a biomarker of lipid peroxidation, were significantly increased in a dose-dependent manner in hepatic and renal tissues of rats following 21 consecutive days of treatment with 2,5-HD when compared with the control values.

### 3.6. Histopathology

**Figure 4:**
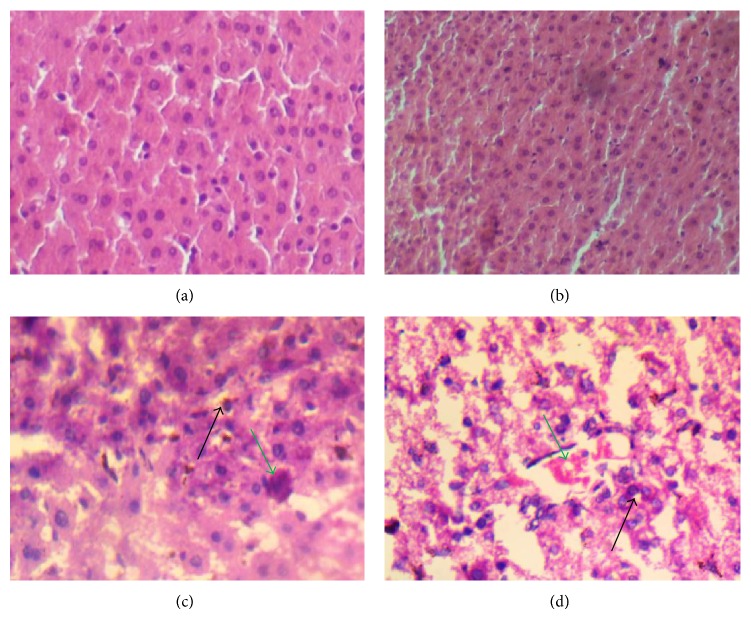
Influence of 2,5-HD on liver morphology after treatment for 21 consecutive days in rats. Representative photomicrographs of liver samples of rats in control (a) and 0.25% 2,5-HD (b) appear normal whereas mild central venous congestion (green arrow) and cellular infiltration (black arrow) by neutrophils were identified in 0.5% 2,5-HD (c) and 1% 2,5-HD (d).

**Figure 5:**
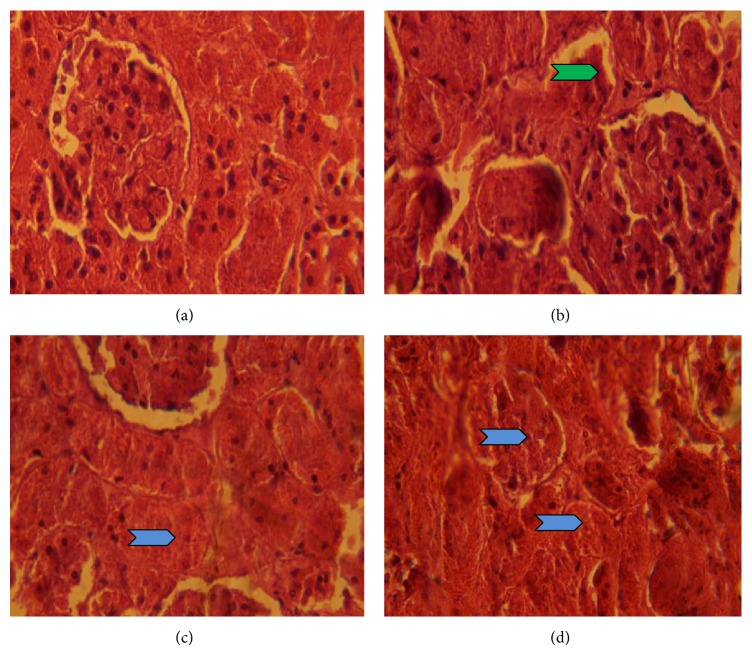
Influence of 2,5-HD on kidney morphology after treatment for 21 consecutive days in rats. Representative photomicrographs of control (a) show normal renal architecture. Kidney histology revealed progressive degeneration of the proximal tubules characterized by mild hemorrhage (green chevron) at interstitium in tubular epithelial cells in 0.25% 2,5-HD (b) and severe vacuolation and renal tubular necrosis (blue chevron) in 0.5% 2,5-HD (c) and 1% 2,5-HD (d).

The representative photomicrographs of the control and 2,5-HD-treated liver and kidney are presented in Figures 4 and 5. The microscopic examination revealed that treatment-related lesions such as mild central venous congestion and cellular infiltration by neutrophils were identified in the liver of rats exposed to 1% 2,5-HD only. Light microscopy showed that the kidneys of control and 0.25% 2,5-HD-treated rats have normal architecture with glomeruli and tubules. However, the morphology of the kidneys of rats exposed to 2,5-HD showed progressive degeneration of the proximal tubules characterized by mild hemorrhage at interstitium in tubular epithelial cells at 0.25% 2,5-HD and severe vacuolation and renal tubular necrosis in 0.5 and 1% 2,5-HD exposed groups.

## 4. Discussion

The current trend in toxicology entails investigating the effects of toxic chemicals at environmentally relevant concentrations, which is the situation normally encountered by the population in industrialized countries. The concentrations of 2,5-HD used in the present study are within the range of human exposures and allow identification of mechanisms of its biological effects on the liver and kidney at low versus high concentrations [29]. The liver and kidney participate in different active biochemical processes involving oxidative metabolism and transport functions, respectively. Some environmental chemicals exert their toxicity through generation of reactive oxygen species (ROS) which are well known to oxidize the biological systems resulting in the pathogenesis of several diseases. The data from the present study revealed that oxidative stress is involved in noxious effects of low concentrations of 2,5-HD on the liver and kidney tissues.

In the present study, exposure to 2,5-HD caused a significant decrease in the body weights but increased the relative liver and kidney weights of the treated rats, thus indicating an overt general and organ toxicity in the rats. Moreover, relative organ weight is an important index of swelling, atrophy, or hypertrophy [30]. An increase in this parameter indicates inflammation while a decrease may be associated with cellular constriction [31]. Hence, the increase in the relative liver and kidney weights of animals exposed to 0.5% and 1% 2,5-HD may suggest swelling/inflammation of the organs. The liver integrity of control and 2,5-HD-treated rats was determined by measuring biochemical markers such as the levels of serum transaminases, alkaline phosphatase, albumin, and bilirubin along with microscopic examination of the organ. Aminotransferases are localized in periportal hepatocytes where they are involved in amino acid metabolism; transamination reactions and their serum activities presumably increase as a result of cellular membrane damage and leakage [32]. The increases in the serum ALT and AST observed in the 2,5-HD-treated rats indicate hepatic damage which may be associated with altered membrane permeability.

Alkaline phosphatase (ALP) is a marker enzyme for the integrity of the hepatobiliary system and the flow of bile into the small intestine. The increase in hepatic ALP activity indicates obstructive event or cholestatic effect following 2,5-HD treatment. Furthermore, the elevation in total and conjugated bilirubin levels observed in the present study indicates posthepatic toxicity possibly caused by an interruption to the drainage of bile in the biliary system following exposure to 2,5-HD. Evaluation of serum albumin level is a good criterion for assessing the secretory ability of the liver [33]. The lack of an effect on the serum albumin levels observed in the experimental animals in the present study suggests that secretory function of the liver was not affected, although there was hepatic injury after 21 days of 2,5-HD treatment. Moreover, alteration in the levels of major lipids such as LDL, HDL, cholesterol, and triacylglycerol could provide useful information on the predisposition to atherosclerosis [34]. Triacylglycerol, LDL, and HDL are associated with lipolysis, transport of plasma cholesterol, and atherosclerotic tendency, respectively. The dose-dependent increases in atherogenic index, serum levels of triacylglycerol, LDL, and cholesterol with a significant decrease in the serum HDL level observed in the 2,5-HD-treated rats in the present investigation may suggest a possible predisposition of the animals to dyslipidemia and other traits of metabolic syndrome associated with cardiovascular diseases.

Kidney damage is associated with decline in renal function which could lead to renal failure. The decrease in renal function evidenced by significant increase in plasma levels of urea and creatinine in rats treated with 2,5-HD was clearly demonstrated in the present investigation. While an increase in serum urea may indicate decrease in reabsorption at the renal epithelium, an increase in serum creatinine reflects impairment in the kidneys, particularly for glomerular filtration rate [10] The data on the renal function parameters observed in the present study following the subchronic exposure of rats to 2,5-HD further corroborated the dose-dependent adverse effect of the xenobiotic. The significant elevation in the serum Na^+^, K^+^, HCO_3_^−^, and Cl^−^ levels is of toxicological significance and may indicate a consequential effect on the ion-dependent processes in the 2,5-HD-treated animals. Based on the serum biochemical parameters presented in the present study, 2,5-HD exposure induced hepatic and renal damages in the treated rats.

The histopathological report revealed that oral exposure to 2,5-HD at environmentally relevant concentrations produced remarkable dose-dependent damaging effect to both liver and kidney of the treated rats, hence supporting the observed biochemical observations. The treatment-related lesions such as mild central venous congestion and cellular infiltration by neutrophils identified in the liver of rats exposed to 2,5-HD suggest its deleterious effect on the structure and function of the liver of the treated animals. Also, the progressive degeneration of the proximal tubules of 2,5-HD-treated animals was characterized by mild hemorrhage at interstitium in tubular epithelial cells with severe vacuolation and renal tubular necrosis. The proximal susceptibility of the tubular epithelium to toxicants has been attributed to the intense filtration of substances from the blood, their transport, and the high energy requirement of these functions [35].

In an attempt to delineate the mechanism of action of 2,5-HD at subcellular levels in the liver and kidney, we investigated its influence on the hepatic and renal antioxidant status. Normally the deleterious effects of oxidative stress are counteracted by the natural antioxidant defense mechanisms to protect the biological system against reactive oxygen species. Antioxidant statuses are regulated by multiple factors. The oxidative status of the cell is the primary factor regulating gene expression and the activity of antioxidant enzymes [36]. Superoxide dismutase (SOD), catalase (CAT), glutathione peroxidase (GPx), and glutathione-S-transferase (GST) are endogenous antioxidant enzymes responsible for the detoxification of deleterious oxygen radicals and their activities are used to assess oxidative stress in cells [9, 37]. The first line of defense to the cells is provided by the existence of a mutually supportive relationship between metalloenzyme SOD, which accelerates the dismutation of endogenous cytotoxic superoxide radicals to H_2_O_2_, and CAT, which converts the deleterious peroxide radicals into water and oxygen [38].

In the present study, the activities of hepatic and renal SOD, CAT, GPx, and GST were markedly increased in rats treated with 2,5-HD. The dose-dependent induction of these antioxidant enzymes may indicate an adaptive response to counter the damaging effect of oxidative stress possibly generated during 2,5-HD metabolism. Glutathione plays a pivotal role in the scavenging of hydroxyl radical and singlet oxygen directly as well as in the detoxification of hydrogen peroxides and lipid hydroperoxides by the activity of GPx. Further, GST is involved in the biochemical conjugation of electrophilic oxidants with GSH to form water-soluble compound products that are readily excreted from the system [25, 39]. The observed decrease in the hepatic and renal GSH level in the present study may suggest an increased demand or overutilization of GSH by the cell possibly to combat ROS generation in the 2,5-HD-treated rats. The deleterious chemical effects of H_2_O_2_ molecules can be divided into the categories of direct activity, originating from their oxidizing properties, and indirect activity in which they serve as a source for more deleterious species, such as hydroxyl radicals and hypochlorous acid [40]. Reactive oxygen species attack cellular components containing polyunsaturated fatty acid residues to produce peroxyl radicals which undergo a cyclization reaction to form endoperoxides and eventually trans-4-hydroxy-2-nonenal and MDA [41]. The dose-dependent increases in the hepatic and renal H_2_O_2_ and MDA levels observed in this study clearly indicate a state of stress in the tissues possibly induced by 2,5-HD or its metabolites.

## 5. Conclusion

The present study obviously demonstrates that an uncontrolled exposure to toxic industrial solvent 2,5-HD is capable of inflicting biological damage leading to the pathology of many conditions including liver and kidney damage. The differential detrimental effects of 2,5-HD on the liver and kidney in rats are strongly associated with biochemical changes including impairment of function, metabolic disorders, oxidative stress, and histological alteration. The data presented herein are novel and show, for the first time, that the hepatorenal toxicity of 2,5-HD in the experimental rats is presumably by increased generation of ROS which led to perturbation of antioxidant defense systems. Hence, the extrapolation of the present animal study to human indicates that 2,5-HD has potential health risk in exposed individuals.

## Figures and Tables

**Table 1:** Body weights and organ weights of rats exposed to 2,5-hexanedione for 21 days.

	Control	0.25% 2,5-HD	0.50% 2,5-HD	1.0% 2,5-HD
Body weight gain (g)	31.56 ± 6.64	12.81 ± 4.54∗	3.71 ± 0.04∗	1.07 ± 3.48∗
RLW (g/100 g bw)	2.27 ± 0.13	2.31 ± 0.11	2.56 ± 0.89∗	2.75 ± 0.93∗
RKW (g/100 g bw)	0.53 ± 0.08	0.54 ± 0.07	0.59 ± 0.04∗	0.71 ± 0.08∗

RLW: relative liver weight; RKW: relative kidney weight. The data are expressed as mean ± SD for eight animals per group. ∗*P* < 0.05 against control.

**Table 2:** Biomarkers of hepatic dysfunction in rats exposed to 2,5-hexanedione for 21 days.

Endpoints	Control	0.25% 2,5-HD	0.50% 2,5-HD	1.0% 2,5-HD
AST (U/L)	21.50 ± 0.71	32.50 ± 0.71∗	39.50 ± 2.89∗	41.08 ± 1.41∗
ALT (U/L)	19.33 ± 1.15	24.50 ± 3.11∗	26.73 ± 1.15∗	27.50 ± 0.71∗
ALP (U/L)	18.33 ± 2.08	23.69 ± 1.15	25.50 ± 4.95∗	29.03 ± 6.68∗
Albumin (g/L)	26.50 ± 1.29	27.10 ± 2.00	28.04 ± 0.82	28.40 ± 1.67
Conjugated bilirubin (mmol/L)	1.75 ± 0.89	1.87 ± 0.53	2.63 ± 0.55∗	2.40 ± 0.55∗
Total bilirubin (mmol/L)	3.75 ± 0.96	5.75 ± 0.96∗	7.20 ± 1.00∗	7.75 ± 1.71∗

ALT: alanine aminotransferase; AST: aspartate aminotransferase; ALP: alkaline phosphatase. The data are expressed as mean ± SD for eight animals per group. ∗*P* < 0.05 against control.

**Table 3:** Lipid profile in male rats exposed to 2,5-hexanedione for 21 days.

Endpoints	Control	0.25% 2,5-HD	0.50% 2,5-HD	1.0% 2,5-HD
HDL (mmol/L)	1.15 ± 0.13	0.88 ± 0.03∗	0.85 ± 0.13∗	0.83 ± 0.12∗
LDL (mmol/L)	0.67 ± 0.19	0.93 ± 0.17∗	1.19 ± 0.28∗	1.35 ± 0.33∗
TG (mmol/L)	1.21 ± 0.14	1.49 ± 0.16∗	1.52 ± 0.13∗	1.57 ± 0.18∗
TC (mmol/L)	1.67 ± 0.13	1.99 ± 0.18∗	2.33 ± 0.21∗	2.37 ± 0.23∗
Atherogenic index	0.58 ± 0.05	1.05 ± 0.08∗	1.40 ± 0.07∗	1.62 ± 0.06∗

HDL: high-density lipoprotein; LDL: low-density lipoprotein; TG: triglyceride; TC: total cholesterol; atherogenic index (LDL/HDL). The data are expressed as mean ± SD for eight animals per group. ∗*P* < 0.05 against control.

**Table 4:** Biomarkers of renal dysfunction in rats exposed to 2,5-hexanedione for 21 days.

Endpoints	Control	0.25% 2,5-HD	0.50% 2,5-HD	1.0% 2,5-HD
Na^+^ (mmol/L)	126.15 ± 1.71	139.67 ± 1.53∗	140.33 ± 2.31∗	147.20 ± 1.41∗
K^+^ (mmol/L)	3.78 ± 0.33	4.03 ± 0.33	4.63 ± 0.61∗	5.05 ± 0.66∗
HCO_3_^−^ (mmol/L)	20.60 ± 1.04	24.70 ± 1.92∗	28.50 ± 1.09∗	34.64 ± 1.13∗
Cl^−^ (mmol/L)	83.30 ± 1.63	88.67 ± 3.21	94.50 ± 1.91∗	96.50 ± 2.22∗
Urea (mmol/L)	14.30 ± 0.62	18.03 ± 1.03∗	18.86 ± 1.67∗	19.46 ± 0.57∗
Creatinine (mmol/L)	89.75 ± 3.30	135.33 ± 3.79∗	151.04 ± 2.00∗	161.80 ± 2.65∗

The data are expressed as mean ± SD for eight animals per group. ∗*P* < 0.05 against control.
